# Brain neurovascular coupling in amyotrophic lateral sclerosis: Correlations with disease progression and cognitive impairment

**DOI:** 10.1111/ene.16540

**Published:** 2024-11-11

**Authors:** Francesca Trojsi, Antonietta Canna, Minoo Sharbafshaaer, Federica di Nardo, Fabrizio Canale, Carla Passaniti, Maria Agnese Pirozzi, Marcello Silvestro, Ilaria Orologio, Antonio Russo, Mario Cirillo, Alessandro Tessitore, Mattia Siciliano, Fabrizio Esposito

**Affiliations:** ^1^ Department of Advanced Medical and Surgical Sciences MRI Research Center, Università degli Studi della Campania Luigi Vanvitelli Naples Italy; ^2^ First Division of Neurology and Neurophysiopathology AOU Università degli Studi della Campania ‘Luigi Vanvitelli’ Naples Italy; ^3^ Montreal Neurological Institute and Hospital, McGill University Montreal Quebec Canada; ^4^ Department of Psychology Università degli Studi della Campania ‘Luigi Vanvitelli’ Caserta Italy; ^5^ Neurosciences Research Centre Molecular and Clinical Sciences Research Institute, St George's, University of London London UK

**Keywords:** amyotrophic lateral sclerosis, cognitive impairment, default mode network, disease progression, neurovascular coupling

## Abstract

**Background and purpose:**

‘Neurovascular coupling’ (NVC) alterations, assessing the interplay between local cerebral perfusion and neural activity within a given brain region or network, may reflect neurovascular unit impairment in amyotrophic lateral sclerosis (ALS). The aim was to explore NVC as a correlation between the functional connectivity and cerebral blood flow within the large‐scale resting‐state functional magnetic resonance imaging brain networks in a sample of ALS patients compared to healthy controls (HCs).

**Methods:**

Forty‐eight ALS patients (30 males; mean age 60.64 ± 9.62 years) and 32 HC subjects (14 males; mean age 55.06 ± 16 years) were enrolled and underwent 3 T magnetic resonance imaging. ALS patients were screened by clinical and neuropsychological scales and were retrospectively classified as very fast progressors (VFPs), fast progressors and slow progressors (SPs).

**Results:**

Neurovascular coupling reduction within the default mode network (DMN) (*p* = 0.005) was revealed in ALS patients compared to HCs, observing, for this network, significant NVC differences between VFP and SP groups. Receiver operating characteristic curve analysis showed that impaired NVC in the DMN at baseline best discriminated VFPs and SPs (area under the curve 75%). Significant correlations were found between NVC and the executive (*r* = 0.40, *p* = 0.01), memory (*r* = 0.32, *p* = 0.04), visuospatial ability (*r* = 0.40, *p* = 0.01) and non‐ALS‐specific (*r* = 0.40, *p* = 0.01) subscores of the Edinburgh Cognitive and Behavioural ALS Screen.

**Conclusions:**

The reduction of brain NVC in the DMN may reflect largely distributed abnormalities of the neurovascular unit. NVC alterations in the DMN could play a role in anticipating a faster clinical progression in ALS patients, aiding patient selection and monitoring during clinical trials.

## INTRODUCTION

Amyotrophic lateral sclerosis (ALS) is the most common and severe form of motor neuron disease, which primarily affects cortical, bulbar and spinal motor neurons, thus causing progressive and irreversible paralysis and atrophy of skeletal muscles [1]. More recently, increasing evidence supports the multi‐system extent of ALS, also accompanied by cognitive and behavioural changes [2] according to a well‐established clinical, genetic and neuropathological continuum with frontotemporal lobar degeneration (FTLD) [3]. Although considerable efforts have been devoted to the understanding of the pathogenesis of ALS, the mechanisms of the disease are still partially unclear and multiple causes could be involved in a ‘multistep’ process of neurodegeneration [4, 5]. Amongst the main pathogenetic hypotheses associated with ALS risk, genetic susceptibility [6], abnormal function of glutamic acid [7], abnormal accumulation of TDP‐43 protein [8], dysfunction of the redox properties of free radicals [9] and depletion of neuroprotective growth factor released by the microvascular networks [10] have been widely investigated. Early vascular cell perturbations and impairment of microvascular function also led to clearance failure of neurotoxic metabolites, further contributing to abnormal phosphorylated TDP‐43 (pTDP‐43) deposits in small cerebral blood vessels in ALS [11]. Elevated levels of pTDP‐43 in motor neurons may also affect microvascular integrity via activation of neurovascular unit (NVU) cells (i.e., vascular cells, glial cells and neurons), inducing neuroinflammation and leakage of the blood–brain barrier (BBB). These pathogenetic pathways may create a self‐reinforcing cycle of vascular malfunction and neurodegeneration [12, 13, 14]. Activation of microglia following BBB leakage may lead to remodelling and degradation of the extracellular matrix in the perineuronal nets, thereby affecting this barrier that protects neurons from oxidative stress and neurodegeneration [15]. This evidence was also recently gathered from preclinical studies performed on animal models of neurodegenerative diseases, including ALS [16, 17].

Magnetic resonance imaging (MRI) may provide the potential to characterize microvascular alterations and vessel supply patterns in a temporo‐spatial manner in vivo, over the course of the neurodegenerative process [18]. However, there is a gap between studies working on understanding disease pathogenesis at the microscopic level and routine clinical MRI studies providing macroscopic information regarding brain structure. This discrepancy is demonstrated by lack of evidence of BBB damage in ALS patients by routine clinical MRI, although microscopic data revealed microvascular alterations and BBB breakdown in rodent models of ALS, beginning before the onset of symptoms and detectable neurodegeneration [19].

Recently, neuroimaging studies of ALS have increased, leading to recognition of brain pathological changes across different stages and phenotypes of disease [20, 21, 22, 23] and to identification of potential MRI indicators useful for predicting ALS progression [24, 25]. However, in ALS there is still no evidence regarding MRI measures of the ‘neurovascular coupling’ (NVC), which quantifies the amount of interplay existing between local cerebral perfusion, evaluated by measures of cerebral blood flow (CBF), and neural activity within a given brain region or network [26]. In particular, the spatial correlation between CBF and a measure of local neural activity has been proposed as a surrogate index at macroscopic level of NVC [26, 27]. Since ALS has been associated with both widespread CBF decrease [23, 28] and functional MRI (fMRI) signal changes during resting state (RS‐fMRI) [29, 30, 31], it is reasonable to examine possible NVC alterations across different RS‐fMRI networks via the spatial combination of individually registered CBF and maps of the amplitude of low‐frequency fluctuations (ALFF), derived, respectively, from arterial spin labelling and RS‐fMRI. With regard to the alterations of RS‐fMRI networks in ALS, most studies reported an impaired functional connectivity in the default mode network (DMN), amongst extra‐motor networks [29, 30, 31]. In particular, by comparing DMN abnormalities in ALS versus FTLD, divergent connectivity patterns have been identified in the two syndromes in early stages of disease, with decreased connectivity in the posterior part of the network in ALS and in the frontal part of the network in FTLD [30], interpreted as an effect of different pathophysiological evolutions within the ALS– FTLD continuum [3, 30].

Thus, in this study, the aim was to explore, for the first time, the potential NVC alterations across different RS‐fMRI networks in a sample of ALS patients in the early stage of disease, monitored for 12 months in comparison to healthy controls (HCs). Additionally, having characterized the cognitive profile and the disease progression of all patients, the NCV data were correlated to cognitive scores and the NVC data at baseline were compared in subsets of patients with different disease progression to identify potential different NCV patterns at ALS onset. It was expected that positive associations would be revealed between reduced cognitive performances and NCV changes in extra‐motor networks and different NCV patterns of abnormalities from baseline by comparing subsets of patients with different disease progression rates.

## MATERIALS AND METHODS

### Subjects

Forty‐eight right‐handed patients (30 males; mean age 60.64 ± 9.62 years), with definite or clinical/laboratory‐supported probable ALS, according to the El Escorial revised criteria [32], showing classic, bulbar, flail limbs or pyramidal phenotypes [33], revealed to be able to tolerate the MRI examination, were consecutively recruited at the First Division of Neurology of the University of Campania ‘Luigi Vanvitelli’ (Naples, Italy) from January 2022 to January 2023. Inclusion criteria were disease onset not earlier than 24 months from enrolment and an age of onset of 40 years or older.

The following clinical features were measured: disability status via the assessment of the ALS Functional Rating Scale Revised (ALSFRS‐R) total score (0–48, with lower total reflecting higher disability) and subscores [34]; pyramidal dysfunction through the evaluation of the UMN score (corresponding to the number of pathological reflexes elicited from 15 body sites) [35]; global cognitive functioning, administering the Italian version of the Edinburgh Cognitive and Behavioural ALS Screen (ECAS) [36, 37]. Disease stage was assessed according to King's clinical staging system [38]. Changes in ALSFRS‐R or ΔALSFRS‐R (ALSFRS‐R from diagnosis to the 12‐month assessment) were calculated based on direct observations. Applying a *k*‐means clustering algorithm [39], a centroid was identified in order to divide the 48 patients into three subgroups: very fast progressors (VFPs) (ΔALSFRS‐R −40.75 ± 3.32, −3.39 points/month, *n* = 8), fast progressors (FPs) (ΔALSFRS‐R −13.85 ± 3.67, −1.15 points/month, *n* = 13) and slow progressors (SPs) (ΔALSFRS‐R −4.26 ± 2.91, −0.55 points/month, *n* = 27).

Genetic analysis was performed for all patients, exploring *C9orf72* repeat expansion and mutations of *SOD1*, *TARDBP* and *FUS/TLS*. No expansions/mutations of these genes were reported.

Thirty‐two right‐handed HC subjects (14 males; mean age 55.06 ± 16.00 years) were enrolled by ‘word of mouth’ and amongst caregivers' friends. They were age, sex and education matched with the enrolled ALS patients and unrelated to them. Moreover, they had no comorbid neurological, psychiatric or medical conditions. They underwent the Mini‐Mental State Examination and their scores were ≥27.

For all subjects, exclusion criteria included medical illnesses or substance abuse that could interfere with cognitive functioning; any other major systemic, psychiatric or neurological diseases; other causes of brain damage, such as lacunae and extensive cerebrovascular disorders shown by fluid‐attenuated inversion recovery (FLAIR) images; and the use of non‐invasive ventilation and a vital capacity of less than 70% of the predicted value.

All participants provided written informed consent to participate in the study according to the Declaration of Helsinki. The study was approved by the Ethics Committee of the University of Campania ‘L. Vanvitelli’ (Protocol no. 591/2018).

### Statistical analysis

Data were processed using IBM SPSS Statistics v. 25 software (IBM, Armonk, NY, USA). The one‐way analysis of variance (ANOVA) and Pearson's chi‐squared test were used to compare the ALS and HC groups on demographic characteristics. The total group of ALS patients was also stratified into three different subgroups according to the disease progression: SPs, FPs and VFPs. These three different subgroups were compared on baseline measures of demographics, clinical and neuropsychological characteristics, and 12‐month follow‐up measures of clinical characteristics. The significance level was set at a Bonferroni corrected *p* value <0.05.

### MRI imaging protocol

Magnetic resonance images were acquired on a 3 T scanner equipped with a 32‐channel parallel head coil (General Electric Healthcare, Milwaukee, WI, USA). The imaging protocol included the following.

- Three‐dimensional T1‐weighted images (gradient‐echo sequence inversion recovery prepared fast spoiled gradient recalled‐echo): repetition time (TR) 6900 ms, echo time (TE) 3.0 ms, resolution 1 × 1 × 1 mm^3^, matrix size 256 × 256, inversion time (TI) 650 ms. Duration 6.49 min.
- 3D‐pseudocontinuous arterial spin‐labelled (PCASL) sequence: TR 5306 ms, TE 10.5 ms, field of view 128 × 128 mm^2^, slice thickness 4 mm, in‐plane resolution 3.8 × 3.8 × 4 mm^3^, post‐labelling delay 2525 ms for a total of 30 subjects, 2025 ms for a total of 40 subjects and 1525 ms for a total of three subjects, 34 slices. Duration 5.09 min.
- Resting‐state echo‐planar imaging sequence: TR 1500 ms, TE 19 ms, field of view 128 × 128 mm^2^, slice thickness 3 mm, 320 time‐points, 44 slices. Duration 8 min.
- FLAIR sequence: TR 11,000 ms, TE 122.7 m, echo train length 18, voxel size 0.5 mm × 0.5 mm × 3 mm, anterior–posterior phase‐encoding direction, number of slices 44. Fat saturation enabled. Duration 3.30 min.

### Image data preprocessing

Standard methods for the preprocessing and analysis of resting‐state functional data as implemented in the Data Processing Assistant for Resting‐State fMRI (DPARSF 6.0) and SPM12 running on MATLAB R2015a were applied (Math‐Works Inc., Natick, MA, USA).

Echo‐planar imaging volumes were corrected for slice timing differences and head motion, band‐pass filtered (0.01–0.1 Hz) and, after alignment to the anatomical T1 volumes, spatially normalized to the Montreal Neurological Institute (MNI coordinates). Last, spatial smoothing with a Gaussian kernel of 6 mm full width at half maximum (FWHM) was applied to the normalized functional data. To reduce the residual effects of head motion (micromotion), as well as the effects of respiratory and cardiac signals, related to heart and respiratory rate, second‐order motion and physiological nuisance correction was performed using a linear regression approach. The regression model included 24 motion‐related predictors (six head motion parameter time series, their first‐order derivatives and the 12 corresponding squared parameter time series) and the mean time courses from a white matter mask and a cerebrospinal fluid mask (as obtained from 3D‐T1w spatial segmentation) as two additional predictors.

Maps of the ALFF, regional homogeneity (ReHo) and degree centrality (DC) were obtained in the MNI space and then a *z*‐transformation was applied to all voxels belonging to a common brain mask in the same standard space and voxel resolution.

Single‐subject whole‐brain CBF maps were calculated from the perfusion weighted imaging and proton density volumes reconstructed from the 3D‐PCASL acquisition based on the calibration formula reported by Alsop et al. [40]. The resulting CBF maps were then aligned to the anatomical T1w images by applying the transformation matrix obtained from the alignment of 3D‐PCASL reference volumes to the corresponding 3D‐T1w images. The aligned CBF maps were then normalized to MNI space using the DARTEL procedure and the internal template derived from the DPARSF pipeline already applied to 3D‐T1w and RS‐fMRI data and resampled to a voxel size of 3 mm × 3 mm × 3 mm. Last, the normalized CBF maps were smoothed with a Gaussian kernel of 6 mm FWHM.

To obtain regional NVC estimates, the standard 100‐region/seven networks parcellation from the Schaefer atlas [41] was first resampled to the 3 mm × 3 mm × 3 mm MNI space using nearest neighbour interpolation. The networks considered in this parcellation were the visual, sensori‐motor, dorsal attention, salience ventral attention, limbic, control networks and the DMN.

Then, for each subject, multiple NVC estimates were computed region‐wise from the Pearson correlation between CBF and ALFF (NVC_ALFF‐CBF_), ReHo (NVC_REHO–CBF_) and DC (NVC_DC–CBF_). The correlation *R* values were computed across all voxels within a region of interest (ROI) or network. For statistical analysis, the *R* values were converted to *z*‐values via Fischer *z* transformation. All *z*‐values were finally organized into two matrices, a 76 (subjects) × 7 (networks) matrix and a 76 (subjects) × 100 (ROIs) matrix.

A linear mixed model was applied to all matrix columns using NVC as a dependent variable, group membership as the independent variable of interest, and age and gender as independent confounding variables (NVC ∼ group + age + gender). False discovery rate (FDR) correction was applied to the series of *p* values expressing the statistical significance of the group membership. The conventional threshold of FDR = 5% was applied to each matrix to detect ROIs or networks exhibiting statistically significant group effects.

The correlation analysis between NVC and all clinical cognitive scores was performed using Pearson correlation. The FDR correction was used for multiple comparisons.

In order to explore the discriminatory power of the MRI metrics between the three subsets of ALS patients, receiver operating characteristics (ROC) were computed through MATLAB implementation analysing the NVC measures that would be significantly different by comparing the ALS subgroups. The ROC curves, area under the curve and its confidence interval (CI) were reported for the MRI metrics found to be the best discriminators between SPs and VFPs, SPs and FPs, and FPs and VFPs.

## RESULTS

### Demographic and clinical assessment

Amyotrophic lateral sclerosis patients and HCs did not differ in demographic, clinical and neuropsychological baseline characteristics (Table 1). Similarly, no statistically significant differences were found between the subgroups of patients with ALS (i.e., SPs, FPs and VFPs) with the same baseline characteristics. Conversely, as for the 12‐month follow‐up, the following were obtained: SPs scored higher than FPs or VFPs in ALSFRS‐R total score, bulbar and respiratory subscores; SPs scored higher than VFPs in ALSFRS‐R total score, bulbar and respiratory subscores; VFPs scored lower than SPs or FPs in upper and lower limb subscores (Table 1). Based on ECAS subscores [36, 37], according to the Strong criteria for frontotemporal spectrum disorder of ALS [42], using the cut‐off according to Siciliano et al. [37], 17 patients had ALS with isolated cognitive impairment. (i.e., 10 with executive function impairment and seven with both executive and language impairments).

**TABLE 1:** Demographic, clinical and neuropsychological characteristics.

Variables	ALS (*n* = 48)	HCs (*n* = 32)	*F*‐test /*χ* ^2^	^a^ *p* value	SPs (*n* = 27)	FPs (*n* = 13)	VFPs (*n* = 8)	*F*‐test /*χ* ^2^	^a^ *p* value	Post hoc (Tukey's HSD or χ^2^)
Baseline measures										
Demographics										
Age, years	60.65 ± 9.62	55.06 ± 16.00	3.79	0.055	59.26 ± 10.18	58.77 ± 7.67	68.37 ± 7.30	3.42	0.041	–
Education, years	10.58 ± 4.11	11.81 ± 3.13	2.05	0.156	11.07 ± 3.75	8.46 ± 4.07	12.38 ± 4.47	2.89	0.066	–
Sex (male)	29 (60.4%)	14 (43.8%)	2.14	0.143	15 (55.6%)	6 (46.2%)	8 (100%)	6.61	0.037	–
Clinical assessment										
Symptom duration, months	16.46 ± 17.26	–	–	–	21.30 ± 21.06	11.00 ± 8.50	9.00 ± 4.62	2.62	0.084	–
UMN burden	8.21 ± 3.82	–	–	–	8.15 ± 4.32	8.31 ± 3.52	8.25 ± 2.81	0.00	0.993	–
King's stage										
Stage 1	22 (45.8%)	–	–	–						
Stage 2	21 (43.8%)	–	–	–						
Stage 3	5 (10.4%)	–	–	–						
ALSFRS‐R total score	41.08 ± 3.29	–	–	–	40.93 ± 3.11	41.62 ± 3.84	40.75 ± 3.32	0.23	0.793	–
ALSFRS‐R subscores										
Bulbar	10.71 ± 1.78	–	–	–	11.19 ± 1.66	9.92 ± 1.75	10.38 ± 1.92	2.51	0.093	–
Upper limbs	9.50 ± 2.63	–	–	–	9.22 ± 2.65	10.15 ± 2.64	9.38 ± 2.72	0.54	0.581	–
Lower limbs	9.25 ± 2.49	–	–	–	8.96 ± 2.45	9.77 ± 2.68	9.38 ± 2.50	0.45	0.635	–
Respiratory	11.69 ± 0.77	–	–	–	11.56 ± 0.93	11.77 ± 0.59	12.00 ± 0.00	1.11	0.336	–
Neuropsychological measures										
ECAS total score	81.04 ± 26.68	–	–	–	92.16 ± 22.34	65.00 ± 21.52	72.38 ± 32.66	6.05	0.005	–
ECAS subscores										
Language	19.67 ± 6.12	–	–	–	21.80 ± 4.90	15.54 ± 6.47	19.75 ± 6.22	5.33	0.009	–
Verbal fluency	13.61 ± 7.55	–	–	–	16.88 ± 6.32	8.15 ± 6.75	12.25 ± 7.44	7.58	0.002	–
Executive functions	24.74 ± 11.14	–	–	–	28.80 ± 10.34	20.46 ± 10.09	19.00 ± 11.26	4.20	0.022	–
Memory	12.57 ± 5.38	–	–	–	13.68 ± 5.13	10.92 ± 4.09	11.75 ± 7.53	1.24	0.298	–
Visuospatial functions	10.43 ± 1.79	–	–	–	10.96 ± 1.24	9.92 ± 2.13	9.63 ± 2.32	2.57	0.088	–
MMSE	–	28.67 ± 1.39	–	–	–	–	–	–	–	–
12‐months’ follow‐up										
ALSFRS‐R total score	28.15 ± 14.02	–	–	–	36.67 ± 4.82	27.77 ± 5.35	0.00 ± 0.00	196.50	**<0.001**	SP > FP***, VFP***; FP > VFP***
ALSFRS‐R subscores		–	–	–						
Bulbar	7.67 ± 4.71	–	–	–	10.63 ± 2.40	6.23 ± 3.63	0.00 ± 0.00	53.52	**<0.001**	SP > FP***, VFP***; FP > VFP***
Upper limbs	6.04 ± 4.29	–	–	–	7.56 ± 3.42	6.62 ± 4.13	0.00 ± 0.00	15.81	**<0.001**	VFP < SP***, FP***
Lower limbs	5.77 ± 3.74	–	–	–	7.56 ± 2.90	5.62 ± 2.69	0.00 ± 0.00	25.95	**<0.001**	VFP < SP***, FP***
Respiratory	8.71 ± 4.38	–	–	–	11.00 ± 1.44	9.31 ± 2.84	0.00 ± 0.00	112.39	**<0.001**	SP > FP*, VFP***; FP > VFP***

*Note*: According to the King's clinical staging system, the number of regions involved gives the stage.

Abbreviations: ALS, amyotrophic lateral sclerosis; ALSFRS‐R, Amytrophic Lateral Sclerosis Functional Rating Scale Revised; ECAS, Edinburgh Cognitive and Behavioural ALS Screen; FPs, fast progressors; HCs, healthy controls; HSD, honestly significant difference; MMSE, Mini‐Mental State Examination; SPs, slow progressors; UMN, upper motor neuron; VFPs, very fast progressors.

^a^ Between‐group differences are reported in bold after Bonferroni correction (for differences between ALS and HCs 0.017 (0.05/3); for differences between SPs, FPs and VFPs 0.002 (0.05/21)).

* Between‐group significant differences.

### Neurovascular coupling data: between‐group comparisons

From the linear mixed model applied to the 100 regions, no statistically significant results were found by comparing the functional parameters that were used to measure the NVC (ALFF, ReHo and DC) in the ALS group versus HC subjects.

**FIGURE 1:**
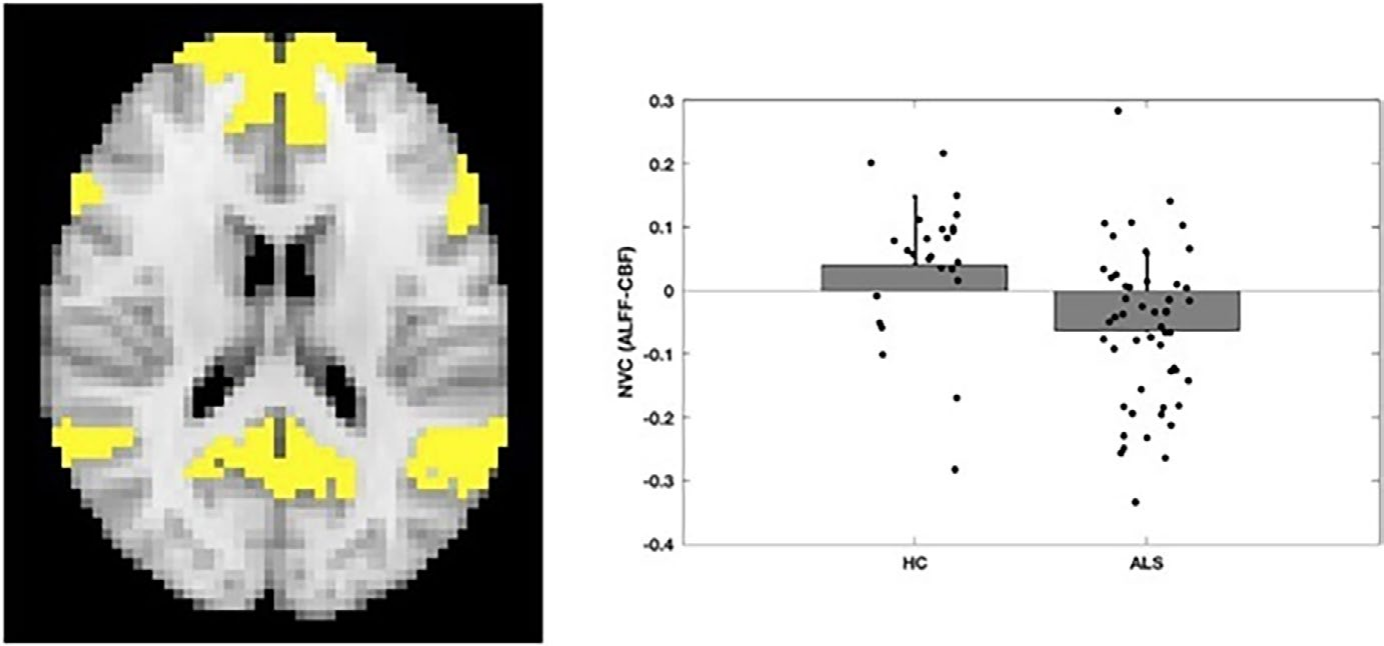
Between‐group comparison of NVC_ALFF‐CBF_ (right plots) in the DMN (left panel): NVC_ALFF‐CBF_ was reduced in ALS patients compared to HCs.

When considering the analysis performed on the seven networks (i.e., visual, sensori‐motor, dorsal attention, salience ventral attention, limbic, control networks and the DMN), a statistically significant difference was found in the DMN (*F* = 8.16, *p* = 0.0056) for the NVC computed as a correlation between the ALFF values and the CBF. No differences were found for the NVC computed as a correlation between ReHo and CBF, nor for the NVC computed as a correlation between DC and CBF. The plot reporting the distribution of the NVC_ALFF‐CBF_ in the DMN value is reported in Figure 1.

In order to evaluate which regions of the DMN mostly contribute to the difference between HCs and ALS patients, the possible differences between HCs and ALS patients in the regions within the DMN were further investigated. However, no regions survived the FDR correction.

**FIGURE 2:**
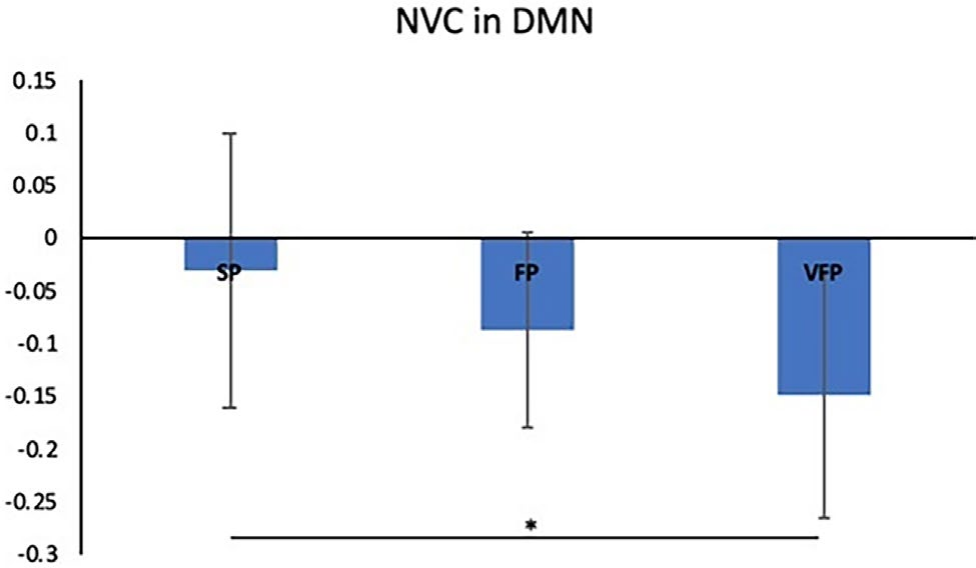
Plots reporting the comparison between the three subsets of patients (SPs, FPs and VFPs).

**FIGURE 3:**
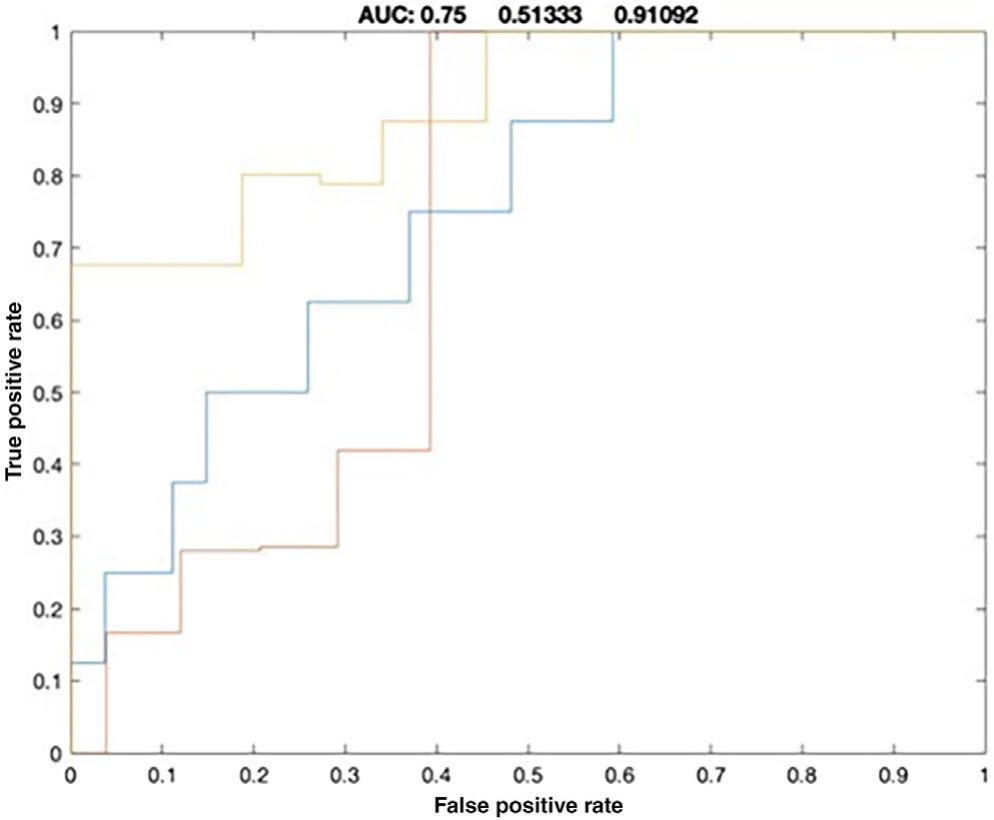
ROC analysis: discriminatory power between SPs and VFPs of NVC_ALFF‐CBF_ in DMN, which showed significant differences between the two subgroups. AUC, area under the curve (orange line).

When comparing the three subsets of ALS patients with each other, significant differences of NVC_ALFF‐CBF_ in DMN were identified by comparing the VFP and SP groups (*p* = 0.03) (Figure 2). Moreover, ROC curve analysis showed that impaired NVC_ALFF‐CBF_ in DMN at baseline best discriminated VFPs and SPs (area under the curve 75%; CI 51.3%–91%) (Figure 3).

### Correlation analysis between NVC_ALFF_ ‐ _CBF_ data in DMN and ECAS subscores

Significant correlations were revealed between NVC_ALFF‐CBF_ in DMN and executive (*r* = 0.40, *p* = 0.01), memory (*r* = 0.32, *p* = 0.04), visuo‐spatial (*r* = 0.40, *p* = 0.01) and no‐ALS‐specific (*r* = 0.4, *p* = 0.01) subscores of ECAS.

## DISCUSSION

In our study, network‐ and region‐based NVC differences were investigated in ALS patients compared to HC subjects. Starting from multiple NVC estimates, derived from the spatial combination of CBF and RS‐fMRI brain maps, it was possible to report a specific pattern of decrease of NVC_ALFF‐CBF_ in DMN in the ALS patients' group. In addition, significant differences in NVC_ALFF‐CBF_ in DMN at baseline were revealed by comparing subsets of patients exhibiting different disease progression rates, allowing the best discrimination between VFP and SP groups. These results may suggest a potential predictive role of NVC measurements on disease progression in ALS. Moreover, significant correlations were revealed between NVC_ALFF‐CBF_ in DMN and executive, memory and visuo‐spatial subscores of ECAS, suggesting a potential association between increased NVC in DMN and cognitive performances in both ALS‐specific and no‐ALS‐specific subscores of ECAS.

NVC_ALFF‐CBF_ in DMN showed a significant decrease in the ALS patients' group and our interpretation of this result is that alterations of NVC measurements in DMN probably reflect an early impairment of NVU integrity in ALS. In fact, in non‐pathological conditions, such as in healthy ageing, densely connected regions such as DMN areas are more metabolically active and may require higher levels of energy consumption [43]. Contrariwise, this coupling appears to be significantly altered in ALS, where the balance between the local connectivity and the obtained metabolic supply is probably disrupted from the early stages, as suggested by the observation of early changes in morphology of neurovascular units in the spinal cord of SOD1^G93A^ mice before evidence of motor neuron degeneration and of neuromuscular denervation [44]. Importantly, with regard to the potential correlation between neurovascular unit disruption and disease progression, using RNA sequencing and immunolabelling on the ventral spinal cord of SOD1^G93A^ mice, Yoshikawa et al. [44] demonstrated that altered morphology of vascular components together with altered expression of microglia and cytotoxic T cell marker genes may contribute to ALS progression. On the other hand, as for the predictive role of DMN abnormalities on ALS progression, in a previous study a significant impairment of functional connectivity within DMN was shown (i.e., in the precuneus), as well as in other areas of motor and extra‐motor resting‐state networks, in FP patients compared to SP ones [25]. These previous findings regarding a main impairment of functional connectivity in both motor and cognitive networks in FP patients, preceding the appearance of structural abnormalities, have been interpreted as a more pronounced and earlier activation of pathogenetic mechanisms, including neuroinflammation, than in SP patients [25]. These results together with evidence of involvement of NVU in ALS pathogenesis and progression [11, 19, 45] may explain the different NVC_ALFF‐CBF_ patterns in DMN observed in different phenotypes of disease. However, future studies involving larger cohorts of ALS patients and using multimodal data (i.e., clinical, genetic, laboratory and MRI data) should be implemented for applying cluster analyses in order to classify patients in different categories, including prognostic ones. Moreover, machine learning methods could be adopted in order to develop prediction models useful at an individual level, to identify distinctive profiles of single individuals and to classify each patient into distinctive prognostic categories [1].

The correlations revealed between NVC_ALFF‐CBF_ in DMN and executive performances (amongst ALS specific subscores of ECAS) and between NVC_ALFF‐CBF_ in DMN and memory, visuo‐spatial and no‐ALS‐specific subscores of ECAS recall recent evidence of NVC dysfunction within DMN related to memory impairment in cerebral small vessel disease [46]. These findings might indicate that the NVC dysfunction of DMN brain regions appears to be present at an early stage of cognitive impairment, considering the mildly impaired cognitive functioning revealed in our population as well as in the study by Li et al. [46]. Moreover, NVC impairment in DMN has been related to cognitive impairment in several pathological conditions, predisposing to cognitive decline through a widespread NVU injury, such as renal disease [47], and diabetes [27, 48]. These findings, in line with our results, may elucidate the interrelationship between pathological features of the cerebral small vessels, NVC dysfunction and cognitive impairment, suggesting the mechanism of cognitive impairment from the perspective of neurovascular dysfunction also in ALS. Probably, in ALS this mechanism could be related to altered functions of TDP‐43 in endothelial cells which may contribute to BBB dysfunction preceding cognitive decline, as recently investigated in animal models [49].

The strength of our study is the use of an advanced approach to processing of MRI data for computing NVC measurements and of a well‐validated neuropsychological tool (i.e., ECAS) in a cohort of ALS patients. However, there are some shortcomings to be acknowledged, including some methodological issues, such as the relatively small sample size, the MRI cross‐sectional study design and the unavailability of multimodal (i.e., clinical, genetic, laboratory and MRI) data as well as of oximetry and partial end‐tidal carbon dioxide measures (amongst factors potentially driving NVC).

## CONCLUSION

Our study supports the hypothesis that the reduction of brain NVC in the DMN may reflect abnormalities of the NVU together with alterations of functional connectivity in extra‐motor areas in non‐demented ALS patients and in VFPs compared to SP patients from early stages of disease. NVC alterations in DMN could play a significant role in the detection of patients presenting faster clinical progression, and this could be critical in patients' selection to attend clinical trials. Finally, evaluating the NVC impairment in DMN could be used as a promising biomarker for monitoring the response to the pharmacological treatments impacting on NVU function, which are also emerging as of interest in ALS.

## AUTHOR CONTRIBUTIONS

**Francesca Trojsi:** Conceptualization; investigation; writing – original draft; methodology; validation; writing – review and editing; project administration; supervision; data curation. **Antonietta Canna:** Conceptualization; investigation; writing – original draft; methodology; validation; visualization; writing – review and editing; software; formal analysis; data curation. **Minoo Sharbafshaaer:** Investigation; writing – review and editing; data curation; writing – original draft. **Federica di Nardo:** Investigation; methodology; validation; software; formal analysis; data curation. **Fabrizio Canale:** Investigation; methodology; validation; software; formal analysis; data curation. **Carla Passaniti:** Investigation; validation; visualization; data curation. **Maria Agnese Pirozzi:** Investigation; methodology; validation; software; formal analysis; data curation. **Marcello Silvestro:** Investigation; formal analysis; validation; data curation. **Ilaria Orologio:** Investigation; validation; formal analysis; data curation. **Antonio Russo:** Investigation; validation; visualization; data curation. **Mario Cirillo:** Investigation; methodology; validation; software; formal analysis; data curation; supervision. **Alessandro Tessitore:** Writing – review and editing; validation; data curation; supervision. **Mattia Siciliano:** Conceptualization; investigation; writing – review and editing; methodology; validation; formal analysis; data curation; supervision. **Fabrizio Esposito:** Conceptualization; funding acquisition; resources; writing – review and editing; validation; methodology; supervision.

## FUNDING INFORMATION

Work was supported by #NEXTGENERATIONEU (NGEU); the Ministry of University and Research (MUR); the National Recovery and Resilience Plan (NRRP); project MNESYS (PE0000006, to NT)—A Multiscale Integrated Approach to the Study of the Nervous System in Health and Disease (DN 1553 11.10.2022).

## CONFLICT OF INTEREST STATEMENT

The authors affirm that there were no financial or commercial affiliations during the research process that could be considered as potential sources of conflict.

## Data Availability

Due to privacy and ethical considerations, the data supporting the findings of this study are not publicly available. Interested researchers may request access to the data by contacting the corresponding author.
